# Estimation of finite population mean for a sensitive variable using dual auxiliary information in the presence of measurement errors

**DOI:** 10.1371/journal.pone.0212111

**Published:** 2019-02-11

**Authors:** Erum Zahid, Javid Shabbir

**Affiliations:** Department of Statistics, Quaid-i-Azam University, Islamabad, Pakistan; Yunnan University of Finance and Economics, CHINA

## Abstract

In this study, we propose a new improved estimator of population mean for the sensitive variable in the presence of measurement error under simple and stratified random sampling. This estimator accounts the auxiliary information as well as the ranks of the auxiliary variable. From theoretical and numerical studies it is shown that a new improved estimator performs better than the existing estimators under study.

## 1 Introduction

In survey sampling, the assumption is made that all the observations are carefully considered on the characteristics under study so the information we obtained is error free. But in practice this assumption is not achieved due to many reasons, including non-response which may arises due to refusal of respondents to give the information or not at home or lack of interest or due to some sensitive issues. In analysis, a basic assumption is that all observations are measured correctly. In multiple regression model, it is assumed that all observations based on the study variable and the auxiliary variable are observed without any error. In many situations these assumptions are violated because of the following reasons. (i) Under the context of qualitative, it is hard to measure some variables (e.g., intelligence, taste, ability, climate, education, poverty etc.). So we use the dummy variables and observations are recorded in terms of values of dummy variables. (ii) In application, some variables are clearly defined but it is hard to take the correct observations (e.g., age is either under reported or over reported in complete year). (iii) It is no doubt that some variables are conceptually defined but is hard to take correct observation on it, instead the observations are taken on closely related variables (e.g., level of education is measured by the number of years of schooling). In all above mentioned cases, it is not possible to obtain true value of the variable. Instead it is recorded with error. So measurement error (ME) appeared because of difference between observed and true value. Also ME is due to the use of imperfect measure of true values of variables. Suppose we are interested to get the average level of anxiety among students, So we take a random sample of some students and measure their level of anxiety. Then we calculate the mean level of anxiety i.e. sample mean. The normality assumption says that if you repeat this process many times and plot the sample means, the distribution will be normal. Usually measurement error and randomized response are studied separately using the known auxiliary or additional information. In reality, when the variable of interest is sensitive, the respondents hesitate to provide the personal information, which gives rise to measurement error.

To estimate the population mean, few researchers discussed the problem of measurement error. [1] discussed some important sources of measurement error in survey data. [2] done the estimation of population mean in the presence of measurement error for ratio-product type estimators. [3] and [4] presented the ratio method of estimation in the presence of measurement error. Further the work is extended by [5]. [6], [7] and [8] studied measurement error and non response together. [9] suggested an estimator for the estimation of population mean in the presence of measurement error and non response under stratified random sampling.

In survey sampling, when the variable of interest is sensitive, then the respondents hesitate to provide their personal information. Direct survey on sensitive question increases the relative bias. [10] introduced the randomized response technique (RRT), which reduces the possible bias and is used to obtain the true information while insuring the privacy of the respondents. For estimation of mean of a sensitive quantitative variable the Randomized Response model (RRM) is extended by [11]. [12] introduced the scrambled randomized response method. [13] proposed the optional RRT method and further this work is extended by [14]. [15] used the scrambled response technique for the estimation of population mean when coefficient of variation is known. [16] used the empirical Bayes estimation for the estimation of sensitive variable. [17] studied the estimation of population mean of sensitive variable in the presence of nonsensitive auxiliary information. [18] and [19] studied the improved estimation of population mean in simple and stratified random sampling.

When the correlation between the study variable and the auxiliary variable is sufficient, then the ranks of the auxiliary variable are also correlated with the study variable and consequently the precision of the estimator increased. [20] suggested the concept of ranks of the auxiliary variable to make efficient estimates. In practice, not much literature has been found in estimating the population mean for the sensitive variable in the presence of measurement error based on dual use of the auxiliary information.

The present paper is organized as: Section 2 gives existing estimators and an improved proposed estimator of population mean for sensitive variable in the presence of measurement error under simple random sampling. Both theoretical and numerical comparison are done in Section 2. In Section 3, some existing estimators and an improved class of estimators is suggested for estimating the finite population mean by incorporating both measurement error and sensitive information simultaneously under stratified random sampling. Efficiency comparison, numerical results and simulation study are also presented in Section 3. Conclusion is given in Section 4.

## 2 Estimators under simple random sampling

Let Ω = Ω_1_, Ω_2_, …, Ω_*N*_ be a finite population of size *N*. Suppose that a simple random sample of size *n* is drawn from Ω by using simple random sampling without replacement. Let *Y* be the sensitive study variable, which is not observed directly and *X* be the non-sensitive auxiliary variable which has positive correlation with *Y*. Let *R*_*x*_ be ranks of the auxiliary variable *X*. Let *S* be a scrambling variable which is independent of *Y* and *X*. We assume that *S* has zero mean and variance $S_{s}^{2}$. The respondent is asked to give a scrambled response for the study variable *Y* given by *Z* = *Y* + *S* and in addition asked to provide a true response for *X*.

Let (*x*_*i*_, *r*_*x*,*i*_, *y*_*i*_, *z*_*i*_) be the observed values and (*X*_*i*_, *R*_*x*,*i*_, *Y*_*i*_, *Z*_*i*_) be the actual values on the variables (*X*, *R*_*x*_, *Y*, *Z*) respectively. Then the measurement errors be *V*_*i*_ = *x*_*i*_ − *X*_*i*_, *U*_*i*_ = *z*_*i*_ − *Z*_*i*_ and *T*_*i*_ = *r*_*x*,*i*_ − *R*_*x*,*i*_. These measurement errors are assumed to be uncorrelated having normal distribution with zero mean and variances $S_{V}^{2}$, $S_{U}^{2}$ and $S_{T}^{2}$ respectively. Let $S_{X}^{2}$, $S_{R_{x}}^{2}$ and $S_{Z}^{2}$ be the population variances; *ρ*_*XZ*_, $\rho_{XR_{x}}$ and $\rho_{ZR_{x}}$ be the coefficients of correlation between their subscripts.

### 2.1 Existing estimators in literature

In this section we consider the following existing estimators.

#### Mean estimator

The usual unbiased mean per unit estimator, is given by

$$\begin{array}{c} {\bar{y}}_{0}=\bar{z}, \end{array}$$ (1)

where $\bar{z}$ is given in Eq (12). The variance of ${\bar{y}}_{0}$, is given by

$$\begin{array}{c} Var\left({\bar{y}}_{0}\right)=\lambda \left(S_{Z}^{2}+S_{U}^{2}\right), \end{array}$$ (2)

where λ = (*n*^−1^ − *N*^−1^).

#### Ratio estimator

The traditional ratio estimator, is given by,

$$\begin{array}{c} {\bar{y}}_{R}=\bar{z}(\frac{\bar{X}}{\bar{x}}), \end{array}$$ (3)

where $\bar{x}$ is the sample mean (see Eq (13)) and $\bar{X}$ is known population mean. The bias and mean square error of ${\bar{y}}_{R}$ to first degree of approximation, are given by

$$\begin{array}{c} B\left({\bar{y}}_{R}\right)≅\frac{\lambda }{\bar{X}}\left[R\left(S_{X}^{2}+S_{V}^{2}\right)-\rho_{XZ}S_{X}S_{Z}\right] \end{array}$$ (4)

and

$$\begin{array}{c} MSE\left({\bar{y}}_{R}\right)≅\lambda \left[\left(S_{Z}^{2}+S_{U}^{2}\right)+R^{2}\left(S_{X}^{2}+S_{V}^{2}\right)-2R\rho_{XZ}S_{X}S_{Z}\right]. \end{array}$$ (5)

where $R=\frac{\bar{Z}}{\bar{X}}$.

#### Difference estimator

The usual difference estimator is given by,

$$\begin{array}{c} {\bar{y}}_{D}=\bar{z}+d\left(\bar{X}-\bar{x}\right), \end{array}$$ (6)

where *d* is the constant, whose value is to be determined optimally. The minimum variance of ${\bar{y}}_{D}$, is given by

$$\begin{array}{c} Var{\left({\bar{y}}_{D}\right)}_{min}=\lambda [S_{Z}^{2}+S_{U}^{2}-\frac{\rho_{XZ}^{2}S_{Z}^{2}S_{X}^{2}}{\left(S_{X}^{2}+S_{V}^{2}\right)}], \end{array}$$ (7)

where optimum value of *d* is $d_{\left(opt\right)}=\frac{\rho_{XZ}S_{Z}S_{X}}{\left(S_{X}^{2}+S_{V}^{2}\right)}$.

#### Khalil estimator

Recently [21] proposed the generalized randomized response estimator, given by,

$$\begin{array}{c} {\bar{y}}_{K}=\left[\bar{z}+k\left(\bar{X}-\bar{x}\right)\right]{\left(\frac{\bar{W}}{\bar{w}}\right)}^{g}, \end{array}$$ (8)

where $\bar{w}=\phi \left(\alpha \bar{x}+\gamma \right)+\left(1-\phi \right)\left(\alpha \bar{X}+\gamma \right)$ and $\bar{W}=\alpha \bar{X}+\gamma$;

*k* and *g* are constants, and *ϕ* is assumed to be an unknown constant which is determined optimally as $\phi =\frac{\rho_{XZ}S_{Z}S_{X}-k\left(S_{X}^{2}+S_{V}^{2}\right)}{gR(S_{X}^{2}+S_{V}^{2})}$. Also *α*(≠0) and *γ* are assumed to be some known parameters of the auxiliary variable *X*. The bias and minimum MSE of ${\bar{y}}_{K}$ to first degree approximation, are given by

$$\begin{array}{c} B\left({\bar{y}}_{K}\right)≅\frac{\lambda }{\bar{Z}}[\frac{g(g+1)}{2}\phi^{2}R^{2}\left(S_{X}^{2}+S_{V}^{2}\right)-g\phi R\rho_{XZ}S_{X}S_{Z}+g\phi kR\left(S_{X}^{2}+S_{V}^{2}\right)] \end{array}$$ (9)

and

$$\begin{array}{c} MSE{\left({\bar{y}}_{K}\right)}_{min}≅\lambda [S_{Z}^{2}+S_{U}^{2}-\frac{\rho_{XZ}^{2}S_{Z}^{2}S_{X}^{2}}{\left(S_{X}^{2}+S_{V}^{2}\right)}], \end{array}$$ (10)

which is exactly equal to the variance of the difference estimator ${\bar{y}}_{D}$, but ${\bar{y}}_{D}$ is preferable over ${\bar{y}}_{K}$ because of unbiasedness.

### 2.2 The proposed estimator

We propose an improved randomized response estimator for estimating the population mean of the sensitive variable, dealing with the problem of measurement error. Measurement error is considered on both the study and the auxiliary variables. A scrambled response of *Y* is observed in form of *Z* = *Y* + *S*, where *S* is distributed as $N(0,S_{s}^{2})$. The proposed estimator, is given by

$$\begin{array}{c} {\bar{y}}_{P}=\bar{z}+m_{1}\left(\bar{X}-\bar{x}\right)\frac{\bar{X}}{\bar{x}}+m_{2}\left({\bar{R}}_{x}-{\bar{r}}_{x}\right)\frac{{\bar{R}}_{x}}{{\bar{r}}_{x}}, \end{array}$$ (11)

where, *m*_1_ and *m*_2_ are constants whose values are to be determined. For obtaining the bias and mean square error, we assume that

$$\begin{array}{c} \delta_{Z}=\sum_{i=1}^{n}\left(Z_{i}-\bar{Z}\right),\delta_{U}=\sum_{i=1}^{n}U_{i}. \end{array}$$

$$\begin{array}{c} \delta_{X}=\sum_{i=1}^{n}\left(X_{i}-\bar{X}\right),\delta_{V}=\sum_{i=1}^{n}V_{i}. \end{array}$$

$$\begin{array}{c} \delta_{R_{x}}=\sum_{i=1}^{n}\left(R_{x_{i}}-{\bar{R}}_{x}\right),\delta_{T}=\sum_{i=1}^{n}T_{i}. \end{array}$$

Adding *δ*_*Z*_ and *δ*_*U*_, we get

$$\begin{array}{c} \delta_{Z}+\delta_{U}=\sum_{i=1}^{n}\left(Z_{i}-\bar{Z}\right)+\sum_{i=1}^{n}U_{i}. \end{array}$$

Dividing both sides by *n*, and then simplifying, we get

$$\begin{array}{c} \bar{z}=\bar{Z}+\frac{1}{n}\left(\delta_{Z}+\delta_{U}\right). \end{array}$$ (12)

Similarly, we can write

$$\begin{array}{c} \bar{x}=\bar{X}+\frac{1}{n}\left(\delta_{X}+\delta_{V}\right), \end{array}$$ (13)

and

$$\begin{array}{c} {\bar{r}}_{x}={\bar{R}}_{x}+\frac{1}{n}\left(\delta_{R_{x}}+\delta_{T}\right). \end{array}$$ (14)

Let $W_{Z}=\frac{\delta_{Z}+\delta_{U}}{n}$, $W_{X}=\frac{\delta_{X}+\delta_{V}}{n}$ and $W_{R_{x}}=\frac{\delta_{R_{x}}+\delta_{T}}{n}$. In order to get the bias and MSE of the proposed estimator, we consider the following relative error terms:

Let $e_{0}=\frac{\bar{z}-\bar{Z}}{\bar{Z}}=\frac{W_{Z}}{\bar{Z}}$, $e_{1}=\frac{\bar{x}-\bar{X}}{\bar{X}}=\frac{W_{X}}{\bar{X}}$, $e_{2}=\frac{{\bar{r}}_{x}-{\bar{R}}_{x}}{{\bar{R}}_{x}}=\frac{W_{R_{x}}}{{\bar{R}}_{x}}$, *E*(*e*_*j*_) = 0, *j* = 0, 1, 2. $E\left(e_{0}^{2}\right)=\frac{\lambda (S_{Z}^{2}+S_{U}^{2})}{{\bar{Z}}^{2}}$, $E\left(e_{1}^{2}\right)=\frac{\lambda (S_{X}^{2}+S_{V}^{2})}{{\bar{X}}^{2}}$, $E\left(e_{2}^{2}\right)=\frac{\lambda (S_{R_{x}}^{2}+S_{T}^{2})}{{\bar{R}}_{x}^{2}}$, $E\left(e_{0}e_{1}\right)=\frac{\lambda \rho_{XZ}S_{X}S_{Z}}{\bar{X}\bar{Z}}$, $E\left(e_{0}e_{2}\right)=\frac{\lambda \rho_{ZR_{x}}S_{Z}S_{R_{x}}}{\bar{Z}\bar{R_{x}}}$ and $E\left(e_{1}e_{2}\right)=\frac{\lambda \rho_{XR_{x}}S_{X}S_{R_{x}}}{\bar{X}\bar{R_{x}}}$

Solving Eq (11) in terms of errors, we have

$$\begin{array}{c} {\bar{y}}_{\left(P\right)}=\bar{Z}\left(1+e_{0}\right)-m_{1}\bar{X}(\frac{e_{1}}{1+e_{1}})-m_{2}{\bar{R}}_{x}(\frac{e_{2}}{1+e_{2}}), \end{array}$$ (15)

Further simplifying, and keeping the terms up to power 2, we have

$$\begin{array}{c} {\bar{y}}_{\left(P\right)}-\bar{Z}≅\bar{Z}e_{0}-m_{1}\bar{X}e_{1}+m_{1}\bar{X}e_{1}^{2}-m_{2}{\bar{R}}_{x}e_{2}+m_{2}{\bar{R}}_{x}e_{2}^{2}, \end{array}$$ (16)

On the lines of [22] and [23], we use the approximation method to derive the MSE of our proposed estimator in simple and stratified random sampling. The signal to noise ratio can easily be obtained by using the expression $C.V=\frac{S.D}{Mean}$. Using above equation the bias of ${\bar{y}}_{\left(P\right)}$, is given by

$$\begin{array}{c} B\left({\bar{y}}_{\left(P\right)}\right)≅m_{1}\frac{\lambda (S_{X}^{2}+S_{V}^{2})}{\bar{X}}+m_{2}\frac{\lambda (S_{R_{x}}^{2}+S_{T}^{2})}{{\bar{R}}_{x}}. \end{array}$$ (17)

Squaring and taking expectation in Eq (16), we have

$$\begin{array}{c} \begin{array}{c} MSE\left({\bar{y}}_{\left(P\right)}\right)≅\lambda \left(S_{Z}^{2}+S_{U}^{2}\right)+m_{1}^{2}\lambda \left(S_{X}^{2}+S_{V}^{2}\right)+m_{2}^{2}\lambda \left(S_{R_{x}}^{2}+S_{T}^{2}\right)\\ +2m_{1}m_{2}\lambda \rho_{XR_{x}}S_{X}S_{R_{x}}-2m_{1}\lambda \rho_{XZ}S_{X}S_{Z}-2m_{2}\lambda \rho_{ZR_{x}}S_{Z}S_{R_{x}}. \end{array} \end{array}$$ (18)

The optimum values of *m*_1_ and *m*_2_ are

$$\begin{array}{c} m_{1(opt)}=\frac{\lambda^{2}\left(\rho_{XR_{x}}S_{X}S_{R_{x}}\right)\left(\rho_{ZR_{x}}S_{Z}S_{R_{x}}\right)-\lambda^{2}\left(S_{R_{x}}^{2}+S_{T}^{2}\right)\left(\rho_{XZ}S_{X}S_{Z}\right)}{\lambda^{2}{\left(\rho_{XR_{x}}S_{X}S_{R_{x}}\right)}^{2}-\lambda^{2}\left(S_{R_{x}}^{2}+S_{T}^{2}\right)\left(S_{X}^{2}+S_{V}^{2}\right)}, \end{array}$$ (19)

and

$$\begin{array}{c} m_{2(opt)}=\frac{\lambda^{2}\left(\rho_{XR_{x}}S_{X}S_{R_{x}}\right)\left(\rho_{XZ}S_{X}S_{Z}\right)-\lambda^{2}\left(S_{X}^{2}+S_{V}^{2}\right)\left(\rho_{ZR_{x}}S_{Z}S_{R_{x}}\right)}{\lambda^{2}{\left(\rho_{XR_{x}}S_{X}S_{R_{x}}\right)}^{2}-\lambda^{2}\left(S_{R_{x}}^{2}+S_{T}^{2}\right)\left(S_{X}^{2}+S_{V}^{2}\right)}, \end{array}$$ (20)

Substitute the optimum values of *m*_1_ and *m*_2_ in Eq (18), we get the minimum MSE of ${\bar{y}}_{P}$, given by

$$\begin{array}{c} MSE{\left({\bar{y}}_{P}\right)}_{min}≅\frac{Q}{\lambda^{2}{\left(\rho_{XR_{x}}S_{X}S_{R_{x}}\right)}^{2}-\lambda^{2}\left(S_{R_{x}}^{2}+S_{T}^{2}\right)\left(S_{X}^{2}+S_{V}^{2}\right)}, \end{array}$$ (21)

where,

$$\begin{array}{cc} Q= & \lambda^{3}\left(S_{Z}^{2}+S_{U}^{2}\right){\left(\rho_{XR_{x}}S_{X}S_{R_{x}}\right)}^{2}+\lambda^{3}\left(S_{R_{x}}^{2}+S_{T}^{2}\right){\left(\rho_{XZ}S_{X}S_{Z}\right)}^{2}\\ & +\lambda^{3}\left(S_{X}^{2}+S_{V}^{2}\right){\left(\rho_{ZR_{x}}S_{Z}S_{R_{x}}\right)}^{2}-\lambda^{3}\left(S_{Z}^{2}+S_{U}^{2}\right)\left(S_{X}^{2}+S_{V}^{2}\right)\left(S_{R_{x}}^{2}+S_{T}^{2}\right)\\ & -2\lambda^{3}\left(\rho_{XZ}S_{X}S_{Z}\right)\left(\rho_{XR_{x}}S_{X}S_{R_{x}}\right)\left(\rho_{ZR_{x}}S_{Z}S_{R_{x}}\right). \end{array}$$

### 2.3 Efficiency comparison

We compare the proposed estimator ${\bar{y}}_{P}$ with respect to ${\bar{y}}_{0},{\bar{y}}_{R},{\bar{y}}_{D}$ and ${\bar{y}}_{K}$, given by

1. From Eqs (2) and (21)
  $MSE{\left({\bar{y}}_{P}\right)}_{min}<Var\left({\bar{y}}_{0}\right)$, if $\frac{Q}{\lambda^{2}{\left(\rho_{XR_{x}}S_{X}S_{R_{x}}\right)}^{2}-\lambda^{2}\left(S_{R_{x}}^{2}+S_{T}^{2}\right)\left(S_{X}^{2}+S_{V}^{2}\right)}-\lambda \left(S_{Z}^{2}+S_{U}^{2}\right)<0$
2. From Eqs (5) and (21)
  $MSE{\left({\bar{y}}_{P}\right)}_{min}<MSE\left({\bar{y}}_{R}\right)$, if $\frac{Q}{\lambda^{2}{\left(\rho_{XR_{x}}S_{X}S_{R_{x}}\right)}^{2}-\lambda^{2}\left(S_{R_{x}}^{2}+S_{T}^{2}\right)\left(S_{X}^{2}+S_{V}^{2}\right)}-\lambda \left(\left(S_{Z}^{2}+S_{U}^{2}\right)+R^{2}\left(S_{X}^{2}+S_{V}^{2}\right)-2R\rho_{XZ}S_{X}S_{Z}\right)<0$
3. From Eqs (7) and (21)
  $MSE{\left({\bar{y}}_{P}\right)}_{min}<Var{\left({\bar{y}}_{D}\right)}_{min}$, if $\frac{Q}{\lambda^{2}{\left(\rho_{XR_{x}}S_{X}S_{R_{x}}\right)}^{2}-\lambda^{2}\left(S_{R_{x}}^{2}+S_{T}^{2}\right)\left(S_{X}^{2}+S_{V}^{2}\right)}-\lambda (S_{Z}^{2}+S_{U}^{2}-\frac{\rho_{XZ}^{2}S_{Z}^{2}S_{X}^{2}}{\left(S_{X}^{2}+S_{V}^{2}\right)})<0$
4. From Eqs (10) and (21)
  $MSE{\left({\bar{y}}_{P}\right)}_{min}<MSE{\left({\bar{y}}_{K}\right)}_{min}$, if $\frac{Q}{\lambda^{2}{\left(\rho_{XR_{x}}S_{X}S_{R_{x}}\right)}^{2}-\lambda^{2}\left(S_{R_{x}}^{2}+S_{T}^{2}\right)\left(S_{X}^{2}+S_{V}^{2}\right)}-\lambda (S_{Z}^{2}+S_{U}^{2}-\frac{\rho_{XZ}^{2}S_{Z}^{2}S_{X}^{2}}{\left(S_{X}^{2}+S_{V}^{2}\right)})<0$

The proposed class of estimator ${\bar{y}}_{P}$ is more efficient than other existing estimators when above conditions 1 to 4 are satisfied.

### 2.4 Numerical results

In this section two populations are generated for simulation study and two are based on real data sets.

#### 2.4.1 Simulation study

We have generated two populations of size 1,000 from multivariate normal distribution with different covariance matrices. The results of simulation is given in Tables 1 and 2. The population means and covariance matrices, are given below:

**Population I**
  $$\begin{array}{c} \mu_{1}=[\begin{array}{c} 500\\ 500\\ 500 \end{array}] \end{array}$$
  and
  $$\begin{array}{c} \sum_{1}=[\begin{array}{ccc} 1000 & 800 & 810\\ 800 & 850 & 820\\ 810 & 820 & 840 \end{array}] \end{array}$$
  *ρ*_*XY*_ = 0.8820, $\rho_{XR_{x}}=0.9702$ and $\rho_{YR_{x}}=0.8884$
**Population II**
  $$\begin{array}{c} \mu_{2}=[\begin{array}{c} 500\\ 500\\ 500 \end{array}] \end{array}$$
  and
  $$\begin{array}{c} \sum_{2}=[\begin{array}{ccc} 400 & 270 & 220\\ 270 & 500 & 300\\ 220 & 300 & 675 \end{array}] \end{array}$$
  *ρ*_*XY*_ = 0.5897, $\rho_{XR_{x}}=0.5105$ and $\rho_{YR_{x}}=0.4965$

**Table 1:** MSE of different estimators for Population I under simulation.

Estimators	MSE without measurement error			MSE with measurement error		
	*n*			*n*		
	100	150	200	100	150	200
${\bar{y}}_{0}$	9.469961	5.962568	4.208872	9.471728	5.963726	4.209671
${\bar{y}}_{R}$	2.102341	1.323696	0.934374	2.106382	1.326300	0.935956
${\bar{y}}_{D},{\bar{y}}_{K}$	2.088259	1.314829	0.928115	2.092113	1.317314	0.929633
${\bar{y}}_{P}$	1.924486	1.211713	0.855327	1.927487	1.213608	0.856596

**Table 2:** MSE of different estimators for Population II under simulation.

Estimators	MSE without measurement error			MSE with measurement error		
	*n*			*n*		
	100	150	200	100	150	200
${\bar{y}}_{0}$	3.732838	2.350305	1.659039	3.734604	2.351463	1.659838
${\bar{y}}_{R}$	3.511012	2.210637	1.560449	3.515051	2.213239	1.562031
${\bar{y}}_{D},{\bar{y}}_{K}$	2.445976	1.540059	1.087100	2.448365	1.541613	1.088114
${\bar{y}}_{P}$	2.250465	1.416959	1.000206	2.252707	1.418413	1.001175

Covariance matrices shows the distribution of sensitive variable *Y*, the auxiliary variable *X* and the ranks of the auxiliary variable *R*_*x*_. There is high correlation in Population I, and weak correlation in Population II. The scrambling response *S* is distributed as *N*(0, 0.01*σ*_*X*_). The response variable is *Z* = *Y* + *S*. We estimate the MSE using *k* = 1000 samples of various sizes selected from each population. Three different sample sizes *n* = 100, 150, 200 are taken from both populations. The expression is given below:

$$\begin{array}{c} MSE\left({\bar{y}}_{i}\right)=\frac{1}{k}\sum_{j=1}^{k}{\left(y_{ij}-\bar{y}\right)}^{2}, \end{array}$$

where *i* = 0, *R*, *D*, *K*, *P*.

Tables 1 and 2 show that the proposed estimator ${\bar{y}}_{P}$ performs better as compared to all other existing estimators for both populations. The MSE of proposed estimators is smaller for Population I as compared to Population II because there is high correlation between the variables in Population I as compared to Population II. As the sample size increases MSE of all the estimators decreases, and it is observed that MSEs of both difference estimator ${\bar{y}}_{D}$ and Khalil estimator ${\bar{y}}_{K}$ is same, but ${\bar{y}}_{D}$ is preferable over ${\bar{y}}_{K}$ because of unbiasedness.

#### 2.4.2 Application to real data

In this section we have considered two data sets for numerical comparisons. Both data sets consist of 654 observations. The data summary is given below (see Tables 3 and 4) and results are given in Tables 5 and 6.

**Table 3:** Data summary.

Variable	Mean	St.Dev	Min	Med	Max	C.V
Forced expiratory volume (*Y*)	2.63	0.86	0.79	2.54	5.79	0.32
Age (*X*)	9.93	2.95	3.00	10.00	19.00	0.29
Smoke (*S*) 0,1	0.10	0.30	0.00	0.00	1.00	3.00

**Table 4:** Data summary.

Variable	Mean	St.Dev	Min	Med	Max	C.V
Forced expiratory volume (*Y*)	2.63	0.86	0.79	2.54	5.79	0.32
Age (*X*)	9.93	2.95	3.00	10.00	19.00	0.29
Sex (*S*)0,1	0.51	0.50	0.00	1.00	1.00	0.98

**Table 5:** MSE of different estimators for Population III under real data.

Estimators	MSE without measurement error			MSE with measurement error		
	*n*			*n*		
	65	100	130	65	100	130
${\bar{y}}_{0}$	0.011658	0.007127	0.005186	0.021543	0.014154	0.010172
${\bar{y}}_{R}$	0.004445	0.002717	0.001977	0.014575	0.009893	0.007074
${\bar{y}}_{D},{\bar{y}}_{K}$	0.004395	0.002687	0.001955	0.014520	0.009859	0.007049
${\bar{y}}_{P}$	0.004364	0.002668	0.001941	0.014412	0.009794	0.007001

**Table 6:** MSE of different estimators for Population IV under real data.

Estimators	MSE without measurement error			MSE with measurement error		
	*n*			*n*		
	65	100	130	65	100	130
${\bar{y}}_{0}$	0.013883	0.008487	0.006175	0.017534	0.010651	0.007757
${\bar{y}}_{R}$	0.008993	0.005498	0.004000	0.012875	0.007820	0.005696
${\bar{y}}_{D},{\bar{y}}_{K}$	0.008606	0.005261	0.003828	0.012426	0.007520	0.005493
${\bar{y}}_{P}$	0.008600	0.005258	0.003825	0.012374	0.007505	0.005467

**Population III** (Source: [24])
**Population IV** (Source: [24])

In both populations the study and the auxiliary variables are identical, but scrambling responses are different. The correlation coefficients for both the Populations are: *ρ*_*XY*_ = 0.7564, $\rho_{XR_{x}}=0.7831$ and $\rho_{YR_{x}}=0.6161$. In Population, III and IV smoke (No = 0, Yes = 1) and sex (Female = 0, Male = 1) are taken as scrambling responses respectively.

Tables 5 and 6 show that the proposed estimator ${\bar{y}}_{P}$ is more efficient as compared to all other considered estimators in both Populations (III and IV). The MSEs of both difference estimator ${\bar{y}}_{D}$ and Khalil estimator ${\bar{y}}_{K}$ are equivalent, but ${\bar{y}}_{D}$ is preferable over ${\bar{y}}_{K}$ because of unbiasedness.

## 3 Estimators under stratified random sampling

Consider a finite population of *N* identifiable units which are partitioned into *L* homogeneous subgroups called strata, such that the *h*^*th*^ strata consist of *N*_*h*_ units, where *h* = 1, 2, …, *L* and $\sum_{h=1}^{L}N_{h}=N$. Let *Y*_*h*_ be the sensitive variable, which do not observe directly and *X*_*h*_ be the non-sensitive auxiliary variable which has a positive correlation with *Y*_*h*_. Let *R*_*x*,*h*_ be the ranks of the auxiliary variable *X*_*h*_ and *S*_*h*_ be a scrambling variable which is independent of *Y*_*h*_ and *X*_*h*_. *S*_*h*_ has zero mean and variance $S_{s_{h}}^{2}$. The respondent is asked to give a scrambled response for the study variable *Y*_*h*_ given by *Z*_*h*_ = *Y*_*h*_ + *S*_*h*_, additionally asked to provide a true response for *X*_*h*_.

A simple random sample of size *n*_*h*_ is drawn without replacement such that $\sum_{h=1}^{L}n_{h}=n$. Let (*x*_*hi*_, *r*_*x*,*hi*_, *y*_*hi*_, *z*_*hi*_) be the observed values and (*X*_*hi*_, *R*_*x*,*hi*_, *Y*_*hi*_, *Z*_*hi*_) be the actual values on the variables (*X*_*h*_, *R*_*x*,*h*_, *Y*_*h*_, *Z*_*h*_) of the *i*^*th*^(*i* = 1, 2, …, *n*) sampled units in the *h*^*th*^ stratum. Then the measurement errors be $V_{hi}^{*}=x_{hi}^{*}-X_{hi}^{*}$, $U_{hi}^{*}=z_{hi}^{*}-Z_{hi}^{*}$ and *T*_*hi*_ = *r*_*x*,*hi*_ − *R*_*x*,*hi*_. These measurement errors are assumed to be uncorrelated and having normal distribution with zero mean and variances $S_{hV}^{2}$, $S_{hU}^{2}$ and $S_{hT}^{2}$ respectively. Let $S_{hX}^{2}$, $S_{hR_{x}}^{2}$ and $S_{hZ}^{2}$ be the population variances; *ρ*_*hXZ*_, $\rho_{hXR_{x}}$ and $\rho_{hZR_{x}}$ be the coefficients of correlation, between their subscripts.

### 3.1 Existing estimators in literature

In this section we consider the following existing estimators.

#### Mean estimator

The usual unbiased mean per unit estimator, is given by

$$\begin{array}{c} {\bar{y}}_{S(0)}=\sum_{h=1}^{L}P_{h}{\bar{z}}_{h}, \end{array}$$ (22)

where $P_{h}=\frac{N_{h}}{N}$ is the known stratum weight and ${\bar{z}}_{h}$ is the mean of the sensitive variable *Z*_*h*_ in the stratum *h*, (see Eq (33)). The variance of ${\bar{y}}_{S(0)}$, is given by

$$\begin{array}{c} Var\left({\bar{y}}_{S(0)}\right)=\sum_{h=1}^{L}P_{h}^{2}\lambda_{h}\left(S_{hZ}^{2}+S_{hU}^{2}\right), \end{array}$$ (23)

where $\lambda_{h}=\left(n_{h}^{-1}-N_{h}^{-1}\right)$.

#### Ratio estimator

The traditional ratio estimator, is given by

$$\begin{array}{c} {\bar{y}}_{S(R)}=\sum_{h=1}^{L}P_{h}\frac{{\bar{z}}_{h}}{{\bar{x}}_{h}}{\bar{X}}_{h}, \end{array}$$ (24)

where ${\bar{X}}_{h}$ is the known population mean and ${\bar{x}}_{h}$ is the sample mean of the auxiliary variable in stratum h, (see Eq (34)). The bias and mean square error of ${\bar{y}}_{S(R)}$, are given by

$$\begin{array}{c} B\left({\bar{y}}_{S(R)}\right)≅\sum_{h=1}^{L}P_{h}\frac{\lambda_{h}}{{\bar{X}}_{h}}\left[R_{h}\left(S_{hX}^{2}+S_{hV}^{2}\right)-\rho_{hXZ}S_{hX}S_{hZ}\right] \end{array}$$ (25)

and

$$\begin{array}{c} MSE\left({\bar{y}}_{S(R)}\right)≅\sum_{h=1}^{L}P_{h}^{2}\lambda_{h}\left[\left(S_{hZ}^{2}+S_{hU}^{2}\right)+R_{h}^{2}\left(S_{hX}^{2}+S_{hV}^{2}\right)-2R_{h}\rho_{hXZ}S_{hX}S_{hZ}\right], \end{array}$$ (26)

where $R_{h}=\frac{{\bar{Z}}_{h}}{{\bar{X}}_{h}}$.

#### Difference estimator

The usual difference estimator, is given by

$$\begin{array}{c} {\bar{y}}_{S(D)}=\sum_{h=1}^{L}P_{h}\left[{\bar{z}}_{h}+d_{h}\left({\bar{X}}_{h}-{\bar{x}}_{h}\right)\right], \end{array}$$ (27)

where *d*_*h*_ is the constant, whose value is to be determined optimally. The minimum variance of ${\bar{y}}_{S(D)}$, is given by

$$\begin{array}{c} Var{\left({\bar{y}}_{S(D)}\right)}_{min}=\sum_{h=1}^{L}P_{h}^{2}\lambda_{h}[S_{hZ}^{2}+S_{hU}^{2}-\frac{\rho_{hXZ}^{2}S_{hZ}^{2}S_{hX}^{2}}{\left(S_{hX}^{2}+S_{hV}^{2}\right)}], \end{array}$$ (28)

where $d_{h(opt)}=\frac{\rho_{hXZ}S_{hZ}S_{hX}}{\left(S_{hX}^{2}+S_{hV}^{2}\right)}$.

#### Khalil randomized response estimator

[21] proposed the estimator, which is given by,

$$\begin{array}{c} {\bar{y}}_{S(K)}=\sum_{h=1}^{L}P_{h}[\left({\bar{z}}_{h}+k\left({\bar{X}}_{h}-{\bar{x}}_{h}\right)\right){\left(\frac{{\bar{W}}_{h}}{{\bar{w}}_{h}}\right)}^{g}], \end{array}$$ (29)

where ${\bar{w}}_{h}=\phi_{h}\left(\alpha_{h}{\bar{x}}_{h}+\gamma_{h}\right)+\left(1-\phi_{h}\right)\left(\alpha_{h}{\bar{X}}_{h}+\gamma_{h}\right)$ and ${\bar{W}}_{h}=\alpha_{h}{\bar{X}}_{h}+\gamma_{h}$;

*k* and *g* are constants, and *ϕ*_*h*_ is assumed to be an unknown constant whose value is to be determined from optimality considerations $\phi_{h}=\frac{\rho_{hXZ}S_{hZ}S_{hX}-k\left(S_{hX}^{2}+S_{hV}^{2}\right)}{gR_{h}\left(S_{hX}^{2}+S_{hV}^{2}\right)}$. Also *α*_*h*_(≠0) and *γ*_*h*_ are assumed to be some known parameters of the auxiliary variable *X*. The bias and minimum MSE of ${\bar{y}}_{S(K)}$, are given by

$$\begin{array}{c} B\left({\bar{y}}_{S(K)}\right)≅\sum_{h=1}^{L}P_{h}\frac{\lambda_{h}}{{\bar{Z}}_{h}}[\frac{g(g+1)}{2}\phi_{h}^{2}R_{h}^{2}\left(S_{hX}^{2}+S_{hV}^{2}\right)-g\phi_{h}R_{h}\rho_{hXZ}S_{hX}S_{hZ}+g\phi_{h}kR\left(S_{X}^{2}+S_{V}^{2}\right)] \end{array}$$ (30)

and

$$\begin{array}{c} MSE{\left({\bar{y}}_{S(K)}\right)}_{min}≅\sum_{h=1}^{L}P_{h}^{2}\lambda_{h}[S_{hZ}^{2}+S_{hU}^{2}-\frac{\rho_{hXZ}^{2}S_{hZ}^{2}S_{hX}^{2}}{\left(S_{hX}^{2}+S_{hV}^{2}\right)}]. \end{array}$$ (31)

which is exactly equal to the variance of the difference estimator ${\bar{y}}_{S(D)}$, but ${\bar{y}}_{S(D)}$ is preferable over ${\bar{y}}_{S(K)}$ because of unbiasedness.

### 3.2 The proposed estimator

An improved randomized response estimator for estimating the population mean of a sensitive variable in the presence of measurement error is proposed. A scrambling response of *Y*_*h*_ is observed in the form of *Z*_*h*_ = *Y*_*h*_ + *S*_*h*_, where *S*_*h*_ is distributed as $N(0,S_{s_{h}}^{2})$. The suggested estimator is given by

$$\begin{array}{c} {\bar{y}}_{S(P)}=\sum_{h=1}^{L}P_{h}[({\bar{z}}_{h}+m_{1h}\left({\bar{X}}_{h}-{\bar{x}}_{h}\right)\frac{{\bar{X}}_{h}}{{\bar{x}}_{h}}+m_{2_{h}}\left({\bar{R}}_{x_{h}}-{\bar{r}}_{x_{h}}\right)\frac{{\bar{R}}_{x_{h}}}{{\bar{r}}_{x_{h}}}], \end{array}$$ (32)

where, *m*_1*h*_ and *m*_2*h*_ are constants whose values are to be determined. ${\bar{R}}_{x_{h}}$ and ${\bar{r}}_{x_{h}}$ are the population mean and sample mean of the ranked of the auxiliary variable, respectively(see Eq (35)). For obtaining the bias and mean square error, we define:

$$\begin{array}{c} \delta_{hZ}=\sum_{i=1}^{n_{h}}\left(Z_{hi}-{\bar{Z}}_{h}\right),\delta_{hU}=\sum_{i=1}^{n_{h}}U_{hi}. \end{array}$$

$$\begin{array}{c} \delta_{hX}=\sum_{i=1}^{n_{h}}\left(X_{hi}-{\bar{X}}_{h}\right),\delta_{hV}=\sum_{i=1}^{n_{h}}V_{hi}. \end{array}$$

$$\begin{array}{c} \delta_{hR_{x}}=\sum_{i=1}^{n_{h}}\left(R_{x,hi}-{\bar{R}}_{x_{h}}\right),\delta_{hT}=\sum_{i=1}^{n_{h}}T_{hi}. \end{array}$$

Adding *δ*_*hZ*_ and *δ*_*hU*_, we get

$$\begin{array}{c} \delta_{hZ}+\delta_{hU}=\sum_{i=1}^{n_{h}}\left(Z_{hi}-{\bar{Z}}_{h}\right)+\sum_{i=1}^{n_{h}}U_{hi}. \end{array}$$

Dividing both sides by *n*_*h*_, and then simplifying, we get

$$\begin{array}{c} {\bar{z}}_{h}={\bar{Z}}_{h}+\frac{1}{n_{h}}\left(\delta_{hZ}+\delta_{hU}\right). \end{array}$$ (33)

Similarly, we can get

$$\begin{array}{c} {\bar{x}}_{h}={\bar{X}}_{h}+\frac{1}{n_{h}}\left(\delta_{hX}+\delta_{hV}\right), \end{array}$$ (34)

and

$$\begin{array}{c} {\bar{r}}_{x_{h}}={\bar{R}}_{x_{h}}+\frac{1}{n_{h}}\left(\delta_{R_{x_{h}}}+\delta_{hT}\right). \end{array}$$ (35)

Let $W_{hZ}=\frac{\delta_{hZ}+\delta_{hU}}{n_{h}}$, $W_{hX}=\frac{\delta_{hX}+\delta_{hV}}{n_{h}}$ and $W_{hR_{x}}=\frac{\delta_{hR_{x}}+\delta_{hT}}{n_{h}}$.

In order to get the bias and MSE of the suggested estimator, we consider the following relative error terms:

Let, $e_{0h}=\frac{{\bar{z}}_{h}-{\bar{Z}}_{h}}{{\bar{Z}}_{h}}=\frac{W_{hZ}}{{\bar{Z}}_{h}}$, $e_{1h}=\frac{{\bar{x}}_{h}-{\bar{X}}_{h}}{{\bar{X}}_{h}}=\frac{W_{hX}}{{\bar{X}}_{h}}$, $e_{2h}=\frac{{\bar{r}}_{x_{h}}-{\bar{R}}_{x_{h}}}{{\bar{R}}_{x_{h}}}=\frac{W_{hR_{x}}}{{\bar{R}}_{x_{h}}}$, *E*(*e*_*jh*_) = 0, *j* = 0, 1, 2. $E\left(e_{0h}^{2}\right)=\frac{\lambda_{h}\left(S_{hZ}^{2}+S_{hU}^{2}\right)}{{\bar{Z}}_{h}^{2}}$, $E\left(e_{1h}^{2}\right)=\frac{\lambda_{h}\left(S_{hX}^{2}+S_{hV}^{2}\right)}{{\bar{X}}_{h}^{2}}$, $E\left(e_{2h}^{2}\right)=\frac{\lambda_{h}\left(S_{hR_{x}}^{2}+S_{hT}^{2}\right)}{{\bar{R}}_{x_{h}}^{2}}$, $E\left(e_{0h}e_{1h}\right)=\frac{\lambda_{h}\rho_{hXZ}S_{hX}S_{hZ}}{{\bar{X}}_{h}{\bar{Z}}_{h}}$, $E\left(e_{0h}e_{2h}\right)=\frac{\lambda_{h}\rho_{hZR_{x}}S_{hZ}S_{hR_{x}}}{{\bar{Z}}_{h}{\bar{R}}_{x_{h}}}$ and $E\left(e_{1h}e_{2h}\right)=\frac{\lambda_{h}\rho_{hXR_{x}}S_{hR_{x}}S_{hX}}{{\bar{X}}_{h}{\bar{R}}_{x_{h}}}$.

Using Eq (32) in terms of errors, we have

$$\begin{array}{c} {\bar{y}}_{S(P)}=\sum_{h=1}^{L}P_{h}[{\bar{Z}}_{h}\left(1+e_{0h}\right)-m_{1h}{\bar{X}}_{h}(\frac{e_{1h}}{1+e_{1h}})-m_{2h}{\bar{R}}_{x_{h}}(\frac{e_{2h}}{1+e_{2h}})]. \end{array}$$ (36)

Further simplifying, and keeping the terms up to power 2, we have

$$\begin{array}{c} {\bar{y}}_{S(P)}-\bar{Z}≅\sum_{h=1}^{L}P_{h}\left[{\bar{Z}}_{h}e_{0h}-m_{1h}{\bar{X}}_{h}e_{1h}+m_{1h}{\bar{X}}_{h}e_{1h}^{2}-m_{2h}{\bar{R}}_{x_{h}}e_{2h}+m_{2h}{\bar{R}}_{x_{h}}e_{2h}^{2}\right]. \end{array}$$ (37)

Using above equation, the bias of ${\bar{y}}_{S(P)}$, is given by

$$\begin{array}{c} B\left({\bar{y}}_{S(P)}\right)≅\sum_{h=1}^{L}P_{h}[m_{1h}\frac{\lambda_{h}\left(S_{hX}^{2}+S_{hV}^{2}\right)}{{\bar{X}}_{h}}+m_{2h}\frac{\lambda_{h}\left(S_{hR_{x}}^{2}+S_{hT}^{2}\right)}{{\bar{R}}_{x_{h}}}]. \end{array}$$ (38)

Squaring and then taking expectations of Eq (37), we have

$$\begin{array}{c} \begin{array}{c} MSE\left({\bar{y}}_{S(P)}\right)≅\sum_{h=1}^{L}P_{h}^{2}[\lambda_{h}\left(S_{hZ}^{2}+S_{hU}^{2}\right)+m_{1h}^{2}\lambda_{h}\left(S_{hX}^{2}+S_{hV}^{2}\right)+m_{2h}^{2}\lambda_{h}\left(S_{R_{x_{h}}}^{2}+S_{hT}^{2}\right)\\ +2m_{1h}m_{2h}\lambda_{h}\rho_{hXR_{x}}S_{hX}S_{hR_{x}}-2m_{1h}\lambda_{h}\rho_{hXZ}S_{hX}S_{hZ}-2m_{2h}\lambda_{h}\rho_{hZR_{x}}S_{hZ}S_{hR_{x}}]. \end{array} \end{array}$$ (39)

From Eq (39), the optimum values of *m*_1*h*_ and *m*_2*h*_ are

$$\begin{array}{c} m_{1h(opt)}=\frac{\lambda_{h}^{2}\left(\rho_{hXR_{x}}S_{hX}S_{hR_{x}}\right)\left(\rho_{hZR_{x}}S_{hZ}S_{hR_{x}}\right)-\lambda_{h}^{2}\left(S_{hR_{x}}^{2}+S_{hT}^{2}\right)\left(\rho_{hXZ}S_{hX}S_{hZ}\right)}{\lambda_{h}^{2}{\left(\rho_{hXR_{x}}S_{hX}S_{hR_{x}}\right)}^{2}-\lambda_{h}^{2}\left(S_{hR_{x}}^{2}+S_{hT}^{2}\right)\left(S_{hX}^{2}+S_{hV}^{2}\right)}, \end{array}$$ (40)

and

$$\begin{array}{c} m_{2h(opt)}=\frac{\lambda_{h}^{2}\left(\rho_{hXR_{x}}S_{hX}S_{hR_{x}}\right)\left(\rho_{hXZ}S_{hX}S_{hZ}\right)-\lambda_{h}^{2}\left(S_{hX}^{2}+S_{hV}^{2}\right)\left(\rho_{hZR_{x}}S_{hZ}S_{hR_{x}}\right)}{\lambda_{h}^{2}{\left(\rho_{hXR_{x}}S_{hX}S_{hR_{x}}\right)}^{2}-\lambda_{h}^{2}\left(S_{hR_{x}}^{2}+S_{hT}^{2}\right)\left(S_{hX}^{2}+S_{hV}^{2}\right)}, \end{array}$$ (41)

Substitute the optimum values of *m*_1*h*_ and *m*_2*h*_ in Eq (39), the minimum MSE ${\bar{y}}_{S(P)}$ is given by

$$\begin{array}{c} MSE{\left({\bar{y}}_{S(P)}\right)}_{min}≅\sum_{h=1}^{L}P_{h}^{2}[\frac{Q_{h}}{\lambda_{h}^{2}{\left(\rho_{hXR_{x}}S_{hX}S_{hR_{x}}\right)}^{2}-\lambda_{h}^{2}\left(S_{hR_{x}}^{2}+S_{hT}^{2}\right)\left(S_{hX}^{2}+S_{hV}^{2}\right)}]. \end{array}$$ (42)

where,

$$\begin{array}{cc} Q_{h}= & \lambda_{h}^{3}\left(S_{hZ}^{2}+S_{hU}^{2}\right){\left(\rho_{hXR_{x}}S_{hX}S_{hR_{x}}\right)}^{2}+\lambda_{h}^{3}\left(S_{hR_{x}}^{2}+S_{hT}^{2}\right){\left(\rho_{hXZ}S_{hX}S_{hZ}\right)}^{2}\\ & +\lambda_{h}^{3}\left(S_{hX}^{2}+S_{hV}^{2}\right){\left(\rho_{hZR_{x}}S_{hZ}S_{hR_{x}}\right)}^{2}-\lambda_{h}^{3}\left(S_{hZ}^{2}+S_{hU}^{2}\right)\left(S_{hX}^{2}+S_{hV}^{2}\right)\left(S_{hR_{x}}^{2}+S_{hT}^{2}\right)\\ & -2\lambda_{h}^{3}\left(\rho_{hXZ}S_{hX}S_{hZ}\right)\left(\rho_{hXR_{x}}S_{hX}S_{hR_{x}}\right)\left(\rho_{hZR_{x}}S_{hZ}S_{hR_{x}}\right). \end{array}$$

### 3.3 Efficiency comparison

The efficiency comparison of ${\bar{y}}_{S(0)},{\bar{y}}_{S(R)},{\bar{y}}_{S(D)}$ and ${\bar{y}}_{S(K)}$ with respect to ${\bar{y}}_{S(P)}$ are given by,

1. From Eqs (23) and (42)
  $MSE{\left({\bar{y}}_{S(P)}\right)}_{min}<Var\left({\bar{y}}_{S(0)}\right)$, if $\sum_{h=1}^{L}P_{h}^{2}[\frac{Q_{h}}{\lambda_{h}^{2}{\left(\rho_{hXR_{x}}S_{hX}S_{hR_{x}}\right)}^{2}-\lambda_{h}^{2}\left(S_{hR_{x}}^{2}+S_{hT}^{2}\right)\left(S_{hX}^{2}+S_{hV}^{2}\right)}]-\sum_{h=1}^{L}P_{h}^{2}\lambda_{h}\left(S_{hZ}^{2}+S_{hU}^{2}\right)<0$
2. From Eqs (26) and (42)
  $MSE{\left({\bar{y}}_{S(P)}\right)}_{min}<MSE\left({\bar{y}}_{S(R)}\right)$, if $\sum_{h=1}^{L}P_{h}^{2}[\frac{Q_{h}}{\lambda_{h}^{2}{\left(\rho_{hXR_{x}}S_{hX}S_{hR_{x}}\right)}^{2}-\lambda_{h}^{2}\left(S_{hR_{x}}^{2}+S_{hT}^{2}\right)\left(S_{hX}^{2}+S_{hV}^{2}\right)}]-\sum_{h=1}^{L}P_{h}^{2}\lambda_{h}\left[\left(S_{hZ}^{2}+S_{hU}^{2}\right)+R_{h}^{2}\left(S_{hX}^{2}+S_{hV}^{2}\right)-2R_{h}\rho_{hXZ}S_{hX}S_{hZ}\right]<0$
3. From Eqs (28) and (42)
  $MSE{\left({\bar{y}}_{S(P)}\right)}_{min}<Var\left({\bar{y}}_{S(D)}\right)$, if $\sum_{h=1}^{L}P_{h}^{2}[\frac{Q_{h}}{\lambda_{h}^{2}{\left(\rho_{hXR_{x}}S_{hX}S_{hR_{x}}\right)}^{2}-\lambda_{h}^{2}\left(S_{hR_{x}}^{2}+S_{hT}^{2}\right)\left(S_{hX}^{2}+S_{hV}^{2}\right)}]-\sum_{h=1}^{L}P_{h}^{2}\lambda_{h}[S_{hZ}^{2}+S_{hU}^{2}-\frac{\rho_{hXZ}^{2}S_{hZ}^{2}S_{hX}^{2}}{\left(S_{hX}^{2}+S_{hV}^{2}\right)}]<0$
4. From Eqs (31) and (42)
  $MSE{\left({\bar{y}}_{S(P)}\right)}_{min}<MSE\left({\bar{y}}_{S(K)}\right)$, if $\sum_{h=1}^{L}P_{h}^{2}[\frac{Q_{h}}{\lambda_{h}^{2}{\left(\rho_{hXR_{x}}S_{hX}S_{hR_{x}}\right)}^{2}-\lambda_{h}^{2}\left(S_{hR_{x}}^{2}+S_{hT}^{2}\right)\left(S_{hX}^{2}+S_{hV}^{2}\right)}]-\sum_{h=1}^{L}P_{h}^{2}\lambda_{h}[S_{hZ}^{2}+S_{hU}^{2}-\frac{\rho_{hXZ}^{2}S_{hZ}^{2}S_{hX}^{2}}{\left(S_{hX}^{2}+S_{hV}^{2}\right)}]<0$

The proposed class of estimators is more efficient than other existing estimators when above Conditions 1 to 4 are satisfied.

### 3.4 Numerical results

In this section two populations are generated for simulation study and one for real data set.

#### 3.4.1 Simulation study

We have generated two populations of size 1,000 from multivariate normal distribution with different covariance matrices. The results are given in Tables 7 and 8. The mean and covariance matrices are give below

**Population V**.
  $$\begin{array}{c} \mu_{5}=[\begin{array}{c} 250\\ 250\\ 250 \end{array}] \end{array}$$
  and
  $$\begin{array}{c} \sum_{5}=[\begin{array}{ccc} 1000 & 800 & 810\\ 800 & 850 & 820\\ 810 & 820 & 840 \end{array}] \end{array}$$
  *N*_1_ = 500 and *N*_2_ = 500,
  *ρ*_1*XY*_ = 0.8554, $\rho_{1XR_{x}}=0.9231$ and $\rho_{1YR_{x}}=0.8705$
  *ρ*_2*XY*_ = 0.8797, $\rho_{2XR_{x}}=0.9725$ and $\rho_{2YR_{x}}=0.8940$
**Population VI**.
  $$\begin{array}{c} \mu_{6}=[\begin{array}{c} 250\\ 250\\ 250 \end{array}] \end{array}$$
  and
  $$\begin{array}{c} \sum_{6}=[\begin{array}{ccc} 675 & 470 & 420\\ 470 & 600 & 400\\ 420 & 400 & 500 \end{array}] \end{array}$$
  *N*_1_ = 400 and *N*_2_ = 600
  *ρ*_1*XY*_ = 0.7172, $\rho_{1XR_{x}}=0.7321$ and $\rho_{1YR_{x}}=0.7104$
  *ρ*_2*XY*_ = 0.7592, $\rho_{2XR_{x}}=0.7496$ and $\rho_{2YR_{x}}=0.7492$

**Table 7:** MSE of different estimators for Population V under simulation.

Estimators	MSE without measurement error			MSE with measurement error		
	*n*_*h*_			*n*_*h*_		
	10%	15%	20%	10%	15%	20%
${\bar{y}}_{S(0)}$	38.3442	17.041870	9.941091	38.366670	17.052020	9.947028
${\bar{y}}_{S(R)}$	9.801616	4.356274	2.541160	9.832481	4.370093	2.549331
${\bar{y}}_{S(D)},{\bar{y}}_{\left(K\right)}$	9.675700	4.300311	2.508515	9.705609	4.313713	2.516432
${\bar{y}}_{S(P)}$	8.422953	3.743535	2.183729	8.445560	3.753748	2.189704

**Table 8:** MSE of different estimators for Population VI under simulation.

Estimators	MSE without measurement error			MSE with measurement error		
	*n*_*h*_			*n*_*h*_		
	10%	15%	20%	10%	15%	20%
${\bar{y}}_{S(0)}$	25.959630	11.537613	6.730274	25.982093	11.547763	6.736212
${\bar{y}}_{S(R)}$	12.978994	5.768442	3.364924	13.009863	5.782262	3.373097
${\bar{y}}_{S(D)},{\bar{y}}_{S(K)}$	11.890829	5.284813	3.082808	11.918461	5.297220	3.090119
${\bar{y}}_{S(P)}$	9.917212	4.407650	2.571129	9.941450	4.418575	2.577538

Covariance matrices show the distribution of sensitive variable *Y*_*h*_, the auxiliary variable *X*_*h*_ and the ranks of the auxiliary variable *R*_*x*,*h*_. Population V consist of two equal strata and Population VI comprises of two unequal strata. In Population V there is high correlation among the variables, and low correlation in Population VI. The scrambling response *S*_*h*_ is distributed as $N(0,0.01\sigma_{X_{h}})$. The response variable is *Z*_*h*_ = *Y*_*h*_ + *S*_*h*_. We estimate the MSE using *k*_*h*_ = 1000 samples of various sizes selected from each strata. Three different sample sizes, 10%, 15% and 20% are taken for both populations. The expression is given below:

$$\begin{array}{c} MSE\left({\bar{y}}_{S(i)}\right)=\frac{1}{k_{h}}\sum_{j=1}^{k_{h}}{\left(y_{hij}-{\bar{y}}_{h}\right)}^{2}. \end{array}$$

where *i* = 0, *R*, *D*, *K*, *P*

Tables 7 and 8 show that the estimator ${\bar{y}}_{S(P)}$ performs better as compared to the estimators ${\bar{y}}_{S(0)}$,${\bar{y}}_{S(R)}$,${\bar{y}}_{S(D)}$ and ${\bar{y}}_{S(K)}$. The efficiency of the estimator ${\bar{y}}_{S(P)}$ is improved when there is sufficient correlation between the study variable and the auxiliary variable. By increasing the sample size, MSE values decreases. As the MSEs of ${\bar{y}}_{S(D)}$ and ${\bar{y}}_{S(K)}$ are equal, so their numerical results are also identical for both the populations.

#### 3.4.2 Application to real data

In this section we consider the real life data set for numerical comparisons. Strata I consist of 318 observations and Strata II contain 336 observations. The data summary is given below (see Tables 9 and 10). The results are given in Table 11. *ρ*_1*XY*_ = 0.7564, $\rho_{1XR_{x}}=0.7831$ and $\rho_{1YR_{x}}=0.6151$

**Table 9:** Data summary strata I.

Variable	Mean	st.Dev	Min	Med	Max	C.V
Forced expiratory volume (*Y*_1_)	2.45	0.65	0.79	2.48	3.83	0.26
Age (*X*_1_)	9.84	2.93	3.00	10.00	19.00	0.29
Smoke (*S*_1_) 0,1	0.12	0.32	0.00	0.00	1.00	2.66

**Table 10:** Data summary strata II.

Variable	Mean	st.Dev	Min	Med	Max	C.V
Forced expiratory volume (*Y*_2_)	2.68	1.00	0.79	2.61	5.79	0.37
Age (*X*_2_)	10.01	2.97	3.00	10.00	19.00	0.29
Sex (*S*_2_)0,1	0.07	0.27	0.00	0.00	1.00	3.85

**Table 11:** MSE of different estimators for Population VII under real data.

Estimators	MSE without measurement error			MSE with measurement error		
	*n*_*h*_			*n*_*h*_		
	10%	15%	20%	10%	15%	20%
${\bar{y}}_{S(0)}$	0.041509	0.027650	0.019442	0.083287	0.055736	0.040301
${\bar{y}}_{S(R)}$	0.015310	0.010116	0.007109	0.058106	0.038821	0.028385
${\bar{y}}_{S(D)},{\bar{y}}_{K}$	0.014450	0.009574	0.006734	0.057221	0.038270	0.027991
${\bar{y}}_{S(P)}$	0.014306	0.009480	0.006667	0.056736	0.037970	0.027792

*ρ*_2*XY*_ = 0.8109, $\rho_{2XR_{x}}=0.7765$ and $\rho_{2YR_{x}}=0.6575$

**Population VII.** (Source: [24])

In Table 11, we observed that the estimator ${\bar{y}}_{S(P)}$ performs better than the estimators ${\bar{y}}_{S(0)}$,${\bar{y}}_{S(R)}$,${\bar{y}}_{S(D)}$ and ${\bar{y}}_{S(K)}$. The estimators ${\bar{y}}_{S(D)}$ and ${\bar{y}}_{S(K)}$ have same MSEs but ${\bar{y}}_{S(D)}$ is preferable due to unbiasedness. As the sample size increases the MSE values decreases, which are the expected results.

## 4 Conclusion

In the present paper, we have proposed a new improved estimator of the finite population mean that encounter additional information on the auxiliary variable as well as on ranks of the auxiliary variable in the presence of measurement error under simple and stratified random sampling. Through simulation study and real life data sets (see Tables 1, 2, 5, 6, 7, 8 and 11) it is observed that the proposed estimators ${\bar{y}}_{P}$ and ${\bar{y}}_{S(P)}$ perform better than the existing estimators, particularly when there is sufficient correlation between the study variable and the auxiliary variable. It is also concluded that difference estimator and [21] estimator are equally efficient, but difference estimator is preferable due to unbiasedness.

## Supporting information

S1 File Data used in the manuscript “S1_File.csv”. (CSV)
